# Calcium binding and voltage gating in Cx46 hemichannels

**DOI:** 10.1038/s41598-017-15975-5

**Published:** 2017-11-20

**Authors:** Bernardo I. Pinto, Amaury Pupo, Isaac E. García, Karel Mena-Ulecia, Agustín D. Martínez, Ramón Latorre, Carlos Gonzalez

**Affiliations:** 1 Centro Interdisciplinario de Neurociencias de Valparaíso, Universidad de Valparaíso, Valparaíso, Chile; 2 Facultad de Odontología, Universidad de Valparaíso, Valparaíso, Chile

## Abstract

The opening of connexin (Cx) hemichannels in the membrane is tightly regulated by calcium (Ca^2+^) and membrane voltage. Electrophysiological and atomic force microscopy experiments indicate that Ca^2+^ stabilizes the hemichannel closed state. However, structural data show that Ca^2+^ binding induces an electrostatic seal preventing ion transport without significant structural rearrangements. In agreement with the closed-state stabilization hypothesis, we found that the apparent Ca^2+^ sensitivity is increased as the voltage is made more negative. Moreover, the voltage and Ca^2+^ dependence of the channel kinetics indicate that the voltage sensor movement and Ca^2+^ binding are allosterically coupled. An allosteric kinetic model in which the Ca^2+^ decreases the energy necessary to deactivate the voltage sensor reproduces the effects of Ca^2+^ and voltage in Cx46 hemichannels. In agreement with the model and suggesting a conformational change that narrows the pore, Ca^2+^ inhibits the water flux through Cx hemichannels. We conclude that Ca^2+^ and voltage act allosterically to stabilize the closed conformation of Cx46 hemichannels.

## Introduction

Connexins (Cxs) are transmembrane proteins that can form two types of non-selective channels, gap junction channels and hemichannels. Gap Junction channels connect the cytoplasm of two adjacent cells and are involved in electrical coupling. Hemichannels, on the other hand, connect the cell interior with the extracellular milieu allowing the influx and release of signaling molecules for autocrine and paracrine cell communication. These proteins control many cellular processes and play an important role in human pathophysiology, as they are expressed in almost every tissue of the human body and are related to several hereditary diseases^1–3^ (Reviewed in^4^).

Cx based channels display two gating mechanisms termed “loop” or “slow” gating and “Vj” or “fast” gating^5^. Both gating mechanisms are regulated by voltage. Each gate is also differentially regulated by several factors. Ca^2+^ and several other polyvalent cations^6–8^ and extracellular pH affect the slow gate^9^, while intracellular pH^10,11^, post-translational modification of the C-terminal domain^8,12^ and metabolic inhibition^13^ modulate the activation of the fast gate. Regulation of Cx hemichannels by extracellular cations has been extensively studied^14–18^. Among these cations, Ca^2+^ is key for the regulation of Cxs. The extracellular media contains Ca^2+^ in the millimolar range, concentrations which have been reported to maintain Cx hemichannels mainly closed at resting potentials^6,16^. Thus, regulation of hemichannels by Ca^2+^ can be important to prevent their opening under physiological conditions, and subsequent leakage of the intracellular content^19^. The affinity of connexins for divalent cations has been measured by analyzing the steady-state currents at different Ca^2+^ concentration and fitting the normalized ionic currents to a Hill equation of inhibition^7,9,16^. Using this methodology, the half maximal inhibitory concentration (IC 50) of Ca^2+^ for Cx32 and Cx26 are 1.3 mM and 0.33 mM, respectively.

Additionally, Ca^2+^ generates a shift in the activation of Cx hemichannels to higher voltages^6,14,18,20^. In the case of Cx46, the effects of voltage on the channel sensitivity to Ca^2+^ and Mg^2+^ was first interpreted as a voltage-dependent block followed by a voltage-dependent stabilization of the blocked state^6^. However, Verselis and Srinivas found that Cx46 hemichannels close even in absence of divalent cations, ruling out the possibility of a divalent block as the gating mechanism, and proposed that external divalent cations stabilize the slow gate closures induced by hyperpolarization^18^. Moreover, AFM imaging of Cx hemichannels shows that in the presence of Ca^2+^ the extracellular mouth of the channel narrows^17,18,21^. Taken together, these results support the hypothesis that voltage-dependent gating is an intrinsic property of the slow gate and divalent cations stabilize the closed state(s). However, in human Cx26 the results indicate that extracellular Ca^2+^ destabilizes the open state of hemichannels facilitating its closure, a mechanism that would require that Ca^2+^ binds to the open state of the channel^16^. A new crystallographic structure of the Cx26 gap junction channel in a Ca^2+^ bound configuration shows no major structural changes, raising the hypothesis of an electrostatic mechanism by which the binding of Ca^2+^ into the pore generates a large and positive electrostatic potential that hinders cation flux^15^. Recently, Lopez and colleagues show that the small charged MTSES (2-sulfonatoethyl methanethiosulfonate) reagent and cadmium ion can pass through the putative Ca^2+^ binding ring^22^, suggesting that the gating of Cx26 hemichannels promoted by Ca^2+^ is not electrostatic. Hence, there are still some contradictions and open questions about the mechanism of Cx hemichannel regulation by extracellular Ca^2+^ and its relation to voltage. Can Ca^2+^ bind to the open state? Is Ca^2+^ binding voltage dependent? Can Ca^2+^ affect the ion flux through an electrostatic mechanism? What is the mechanism through which Ca^2+^ is stabilizing the closed states? To address these issues we tested the effects of voltage and Ca^2+^ in Cx46 hemichannels expressed in *X. laevis* oocytes. We found that Cx46 apparent affinity for Ca^2+^ changes with voltage, reaching a constant value at voltages that keep most of the channels closed and decreasing exponentially as we get to higher voltages. These observations can only be explained by a voltage-independent binding to the closed state by Ca^2+^. Further analysis of the channel kinetics reveals two distinguishable kinetic steps and these are directly regulated by voltage, with Ca^2+^ accelerating the deactivation kinetics which reaches a constant value at high Ca^2+^ concentrations. This cannot be explained by a linear mechanism and indicate that Ca^2+^ binding is allosterically coupled to the voltage sensor. The best kinetic model able to account for the equilibrium and kinetic data considers three voltage dependent steps and six independent Ca^2+^ binding sites. Analysis of the water permeability of Cx46 in hypotonic medium and under Ca^2+^-free and high Ca^2+^ conditions we found that Ca^2+^ binding to the Cx46 channel promoted a substantial reduction in the passage of water indicating that Ca^2+^ promotes the closing of a physical gate in the channel. This work supports that Ca^2+^ binds only to the closed state of the channel by a voltage independent mechanism. Remarkably, a detailed analysis of the activation and deactivation channel kinetics suggests that Ca^2+^ and voltage sensors are allosterically coupled to promote Cx46 hemichannel closing.

## Results

### Regulation of Cx46 hemichannels by calcium and voltage

**Figure 1:**
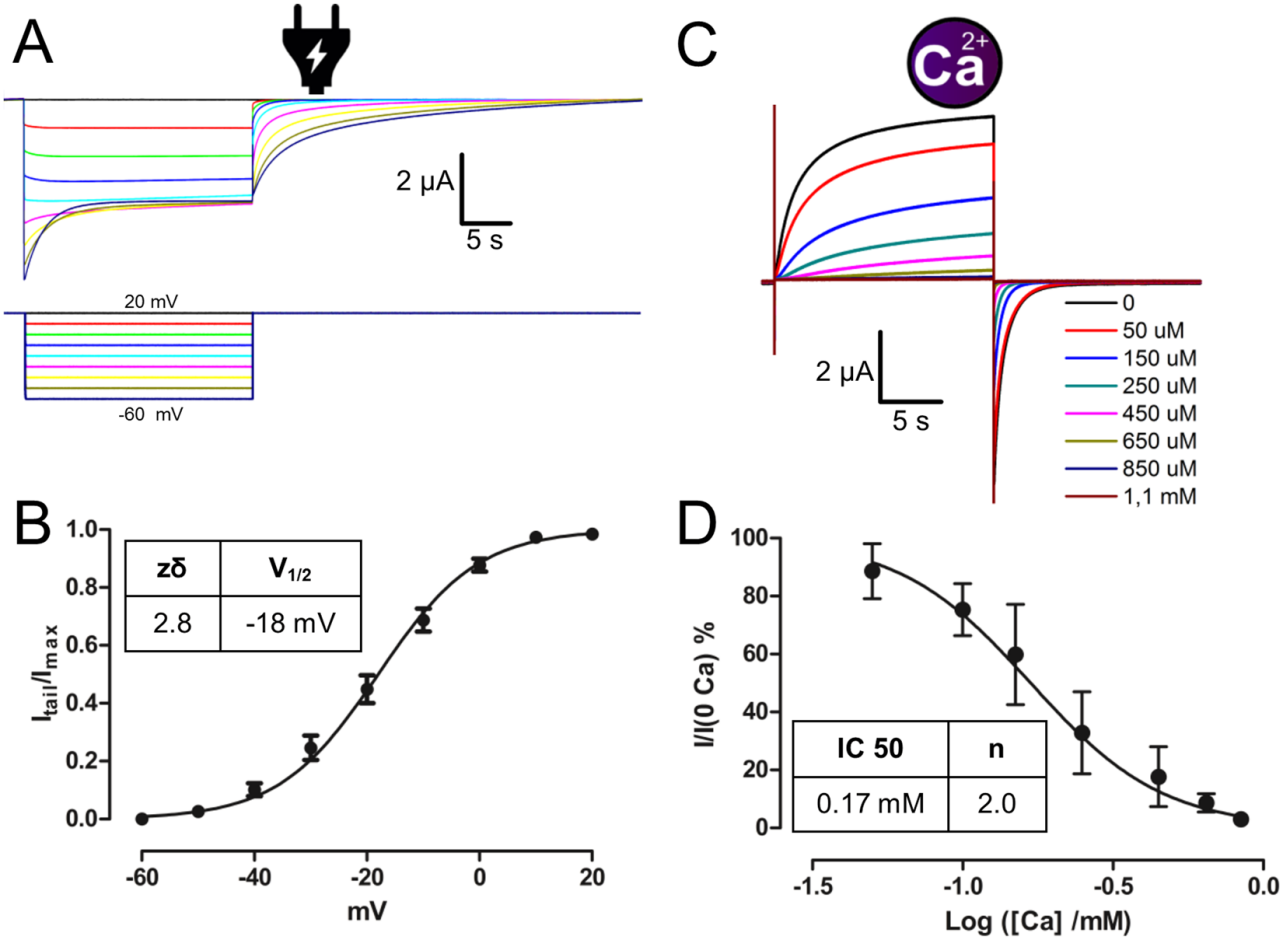
Regulation of Cx46 hemichannels by voltage and calcium. (**A**) Oocytes expressing Cx46 maintained at a holding potential of +20 mV were subjected to 8 hyperpolarizing pulses from 20 mV to −60 mV in 10 mV steps. Current traces that correspond to voltage traces have the same color. (**B**) Graph depicting the voltage activation curve of Cx46 obtained from tail currents. The solid line represents the fitting of the data to equation (1) and using the parameters given in the inset table. (**C**) Oocytes at a holding potential of −70 mV were subjected to depolarizing pulses to 0 mV at different Ca^2+^ concentrations (from 0 to 1.1 mM). The colored line indicates the concentration of Ca^2+^ present in the bath. (**D**) Graph depicting the normalized tail currents at the different Ca^2+^ concentration. The solid line represents the fitting of the data using equation (2) and the parameters given in the inset table.

The voltage regulation of Cx46 can be evidenced from changes in the conductance observed when hyperpolarizing pulses are applied from a holding potential of 20 mV (Fig. 1A)^6^. The Cx46 hemichannel voltage dependence can be obtained by measuring the normalized tail current right after the pulse. We obtained the (*I*_*tail*_ *(V)/I*_*max*_) -voltage relationship and fitted this data to a Boltzmann function (equation 1):

1 $$\frac{{I}_{tail}(V)}{{I}_{\max }}=\frac{1}{1+{e}^{-z\delta (V-{V}_{1/2})/RT}}$$

with a half-maximal voltage (*V*_*1/2*_) of −18 mV and an apparent number of gating charges (*zδ*) of 2.8 elementary charges (Fig. 1B). The regulation of Cx46 by Ca^2+^ can be readily observed by giving a voltage pulse from −70 mV to 0 mV at different Ca^2+^ concentrations ([Ca]). As the Ca^2+^ concentration in the bath increases, the current induced by the depolarizing pulse decreases (Fig. 1C). The normalized current (*I*_*Ca*_ */I*_*Ca*=0_) at the end of the pulse can be fitted using the Hill equation for inhibition (equation 2):

2 $$I(Ca)/I(0Ca)=\frac{1}{1+{(\frac{[C{a}^{+2}]}{IC50})}^{n}}$$

with a *IC50* of 0.17 mM and a Hill slope (*n)* of 2 (Fig. 1D).

### Calcium inhibition of Cx46 is modified by voltage

**Figure 2:**
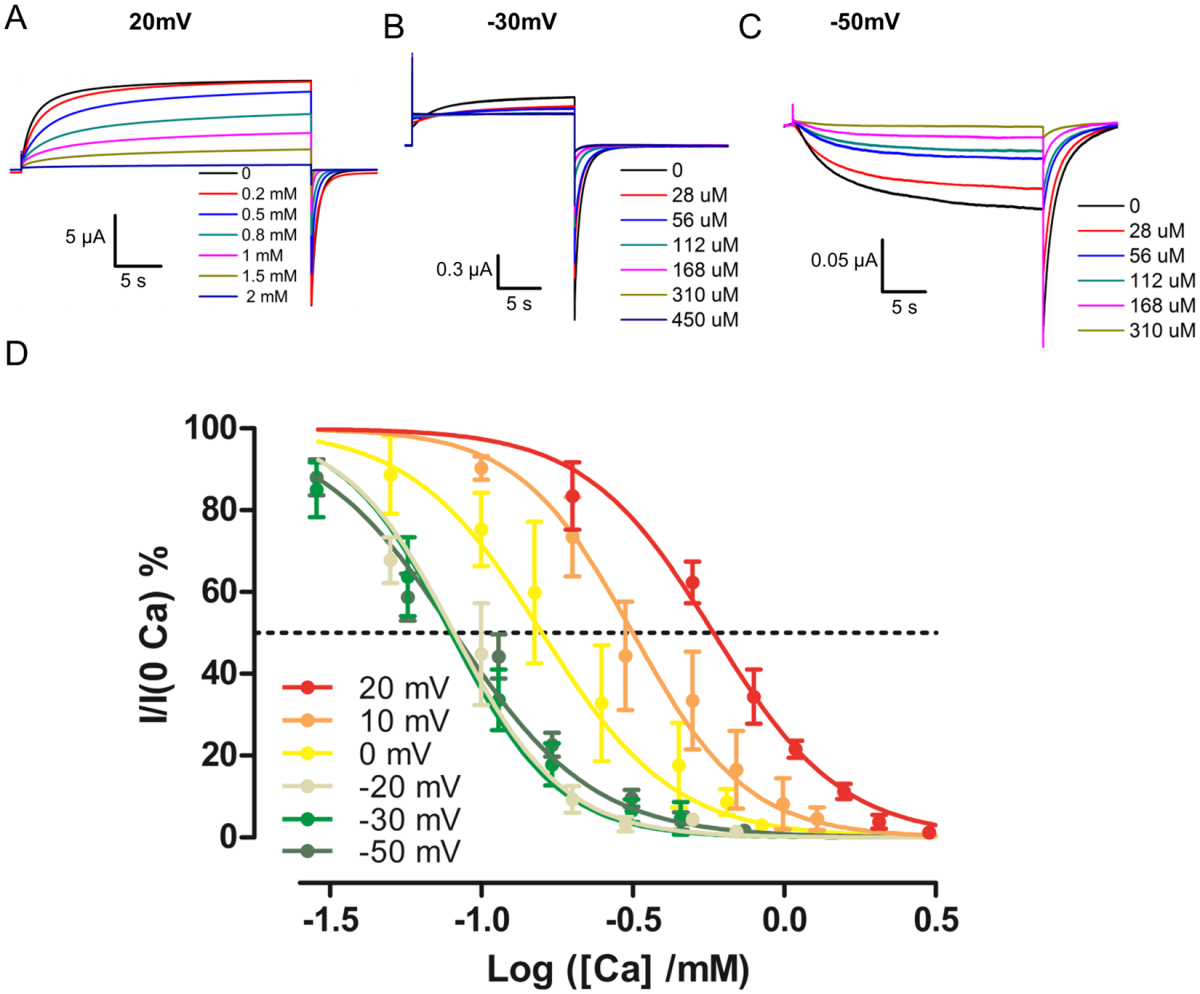
Inhibition of Cx46 hemichannels by calcium at different voltages. Oocytes at a holding potential of −70 mV were subjected to depolarizing pulses of (**A**) 20 mV, (**B**) −30 mV and (**C**) −50 mV. At each voltage different Ca^2+^ concentrations were tested. The colored line indicates the concentration of Ca^2+^ present in the bath. (**D**) Ca^2+^ inhibition curves for the different voltages used. Data presented as mean ± SEM, the lines indicate the fit to the Hill equation (Table 1).

We measured the apparent affinity of Cx46 hemichannels evaluating the degree of current inhibition promoted by different voltages and Ca^2+^ concentrations. A squared pulse at different voltages: 20, 10, 0, −20, −30 and −50 mV was applied from a holding potential of −70 mV for 20 or 30 s (Fig. 2A–C). Tail currents at −70 mV were measured after the voltage steps. The relative current vs. Ca^2+^ concentration curve was obtained. Increasing the pulse duration did not affect these Ca^2+^ inhibition curves. For each test voltage the tail current vs. Ca^2+^ concentration data were fitted using a Hill equation (equation 2) and the Log IC50 and the Hill slope was determined. Increasing the test voltage increased the apparent half inhibitory concentration of Ca^2+^ without noticeable effects on the Hill slope (Fig. 2D and Table 1).

**Table 1:** Apparent Ca^2+^ affinity and Hill slope of Cx46 at different voltages. Data presented as mean ± SEM. N; number of inhibition curves.

Voltage	−50 mV	−30 mV	−20 mV	0 mV	10 mV	20 mV
Log *IC50*	−1.09 ± 0.06	−1.1 ± 0.1	−1.09 ± 0.06	−0.8 ± 0.1	−0.5 ± 0.1	−0.2 ± 0.1
Hill slope	−1.9 ± 0.2	−2.5 ± 0.7	2.4 ± 0.2	−2.0 ± 0.2	−2.3 ± 0.2	−2.0 ± 0.6
N	6	6	4	5	3	3

### Stabilization of the closed state by Ca^2+^ accounts for the changes in affinity with voltage

The increase in the affinity for Ca^2+^ as voltage decreased can be explained either by a voltage dependent block or by a stabilization of the closed state. It has been shown that the channel can close in the absence of divalent cations and that extracellular divalent cations promote an stabilization of the closed channel^18^. This strongly argues against a voltage dependent block of the open state. To explain the changes in affinity we developed several linear models in which a sequential binding of Ca^2+^ to the closed state of Cx46 helps to stabilize the closed conformation.

R1 $${\rm{O}}\,\mathop{\longleftrightarrow }\limits^{{{\rm{K}}}_{{\rm{V}}}}\,{\rm{C}}\,\mathop{\longleftrightarrow }\limits^{{\rm{K1}}}\,{\rm{CCa}}\,\mathop{\longleftrightarrow }\limits^{{\rm{K2}}}\,{{\rm{CCa}}}_{{\rm{2}}}\cdot \cdot \cdot \cdot {{\rm{CCa}}}_{n-1}\,\mathop{\longleftrightarrow }\limits^{{\rm{Kn}}}\,{{\rm{CCa}}}_{{\rm{n}}}$$

In kinetic scheme R1 only the closed-open transition, defined by *K*_*V*_ is voltage-dependent; depolarizing voltages favor the *O* state, hence the apparent Ca^2+^ affinity of this model should decrease as the voltage increases. All the other states are reached through the sequential binding of Ca^2+^ and defined by the several dissociation constants, *K1*, *K2*, *Kn*. (See Appendix 1). Alternatively, we can also use a linear model consisting of three states O-C-CCa_n_ where Ca^2+^ binding to the closed state is described by a Hill equation. The Hill equation assumes highly cooperative binding steps. We fitted all the inhibition curves to these models and the resulting fitting parameters determined for three of them are presented in Table 2.

**Table 2:** Parameters of inhibition curves fitted using three linear models for Ca^2+^ binding. The parameters estimate and the errors associated are presented.

Model	Parameter	Estimate	Standard Error
Linear - Hill	LogKd	−2.31	0.07
	n	1.99	0.07
Linear - 2 sites	LogKd1	0.0	1.1
	LogKd2	−2.4	1.2
Linear - 3 sites	LogKd1	0	1.4
	LogKd2	−2	1.5
	LogKd3	7	1.5 * 10^6^

It can be seen that the linear model using the Hill equation has the lowest errors in the parameters estimates compared to the other two linear sequential binding models. Increments in the number of parameters are associated with the error increase, as can be observed for the three binding sites model. Also, the linear model with two binding sites resembles the linear model using the Hill equation. In the two sites model the second binding has an affinity about 250 times greater than the first, thus binding to the first site will generate a highly cooperative binding to the second site. These are the reasons why the linear model using the Hill equation was preferred over the alternative linear models.

**Figure 3:**
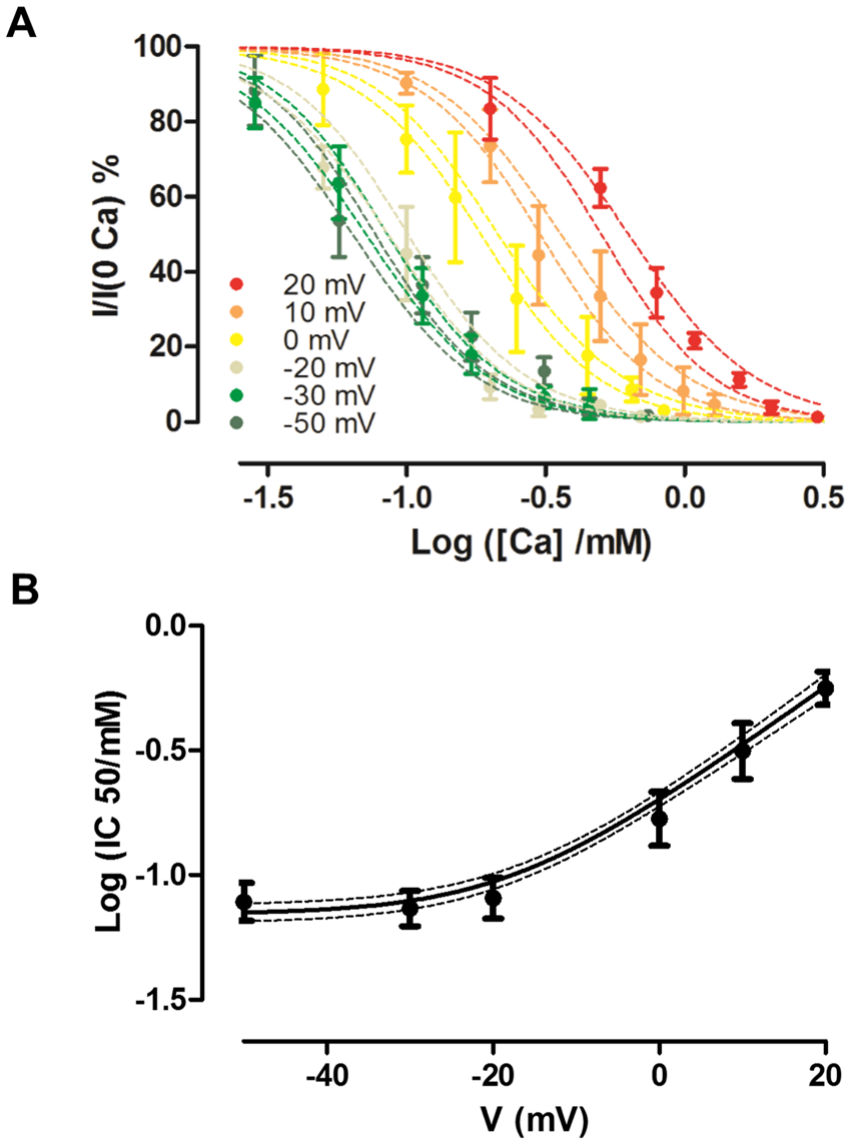
Stabilization of the closed state reproduces the observed changes in inhibition. (**A**) Ca^2+^ inhibition data at different voltages was fitted using the linear Hill model. The discontinuous lines show the 95% confidence intervals of the model for the inhibition curves obtained at different voltages. (**B**) The points show the Log *IC50* determined at different voltages. The black line indicates the prediction of the Log *IC50* according to the linear Hill model, and the dotted lines show the 95% confidence intervals.

The proposed model reproduces the effects of voltage over Ca^2+^ affinity (Fig. 3A). As it was found experimentally, the model predicts that the Log (*IC50)* reaches a constant value of (1/*n*) Log (*K*_*D*_ *)* when *V* ≪*V*_*1/2*_ and that the Log (*IC50*) increases linearly with voltage when *V*≫ *V*_*1/2*_ (Fig. 3B), as expected from the equation below (equation s16 in Appendix 1):

$$\mathrm{log}(IC50)=\frac{1}{n}\,\mathrm{log}({K}_{D}(1+{e}^{-z\delta (V-{V}_{1/2})/RT}))$$

Moreover, we tested alternative models for calcium inhibitions that are shown in Supplementary Figure 1. Contrary to our model and experimental data, a voltage dependent Ca^2+^ binding generates non-saturating Log *IC50* curve (Suppl. Figure 1A), the binding of Ca^2+^ to the open state produces an incomplete inhibition of the current and saturation of Log *IC50* at higher voltages (Suppl. Figure 1B), a blocking mechanism increase the Log *IC50* at higher voltages (Suppl. Figure 1C) and Ca^2+^ binding to intermediate states generates a non-saturating Log *IC50* curve (Suppl. Figure 1D). The failure of all these models to reproduce the experimental data indicates that Ca^2+^ binds only to the last closed state and this binding is not voltage dependent.Thus, the voltage dependence of Ca^2+^ inhibition arises solely from the intrinsic voltage dependence of the gating mechanism.

Our model shows that using a voltage pulse that keeps most of the channels closed will result in an inhibition curve that resembles that of the closed state. Thus, the apparent affinity for the closed state (*Kd*) can be determined by two methods. First, we can determine the voltage dependence of the channel and later the affinity for Ca^2+^ at any voltage. With this data, we can extrapolate the *Kd* of the closed state. Second, we can determine the affinity of the channel using very low voltages that will keep most of the channels closed. Our results using the extrapolation from 0 mV gives a *Kd* of 3 µM and using the IC 50 at -50 mV a *Kd* of 8 µM. By fitting all the data points we obtain a *Kd* of 5 µM (95% CI: 3.9–6.8) (See Appendix 1 for details).

### Role of voltage and Ca^2+^ in the kinetics of the channel

**Figure 4:**
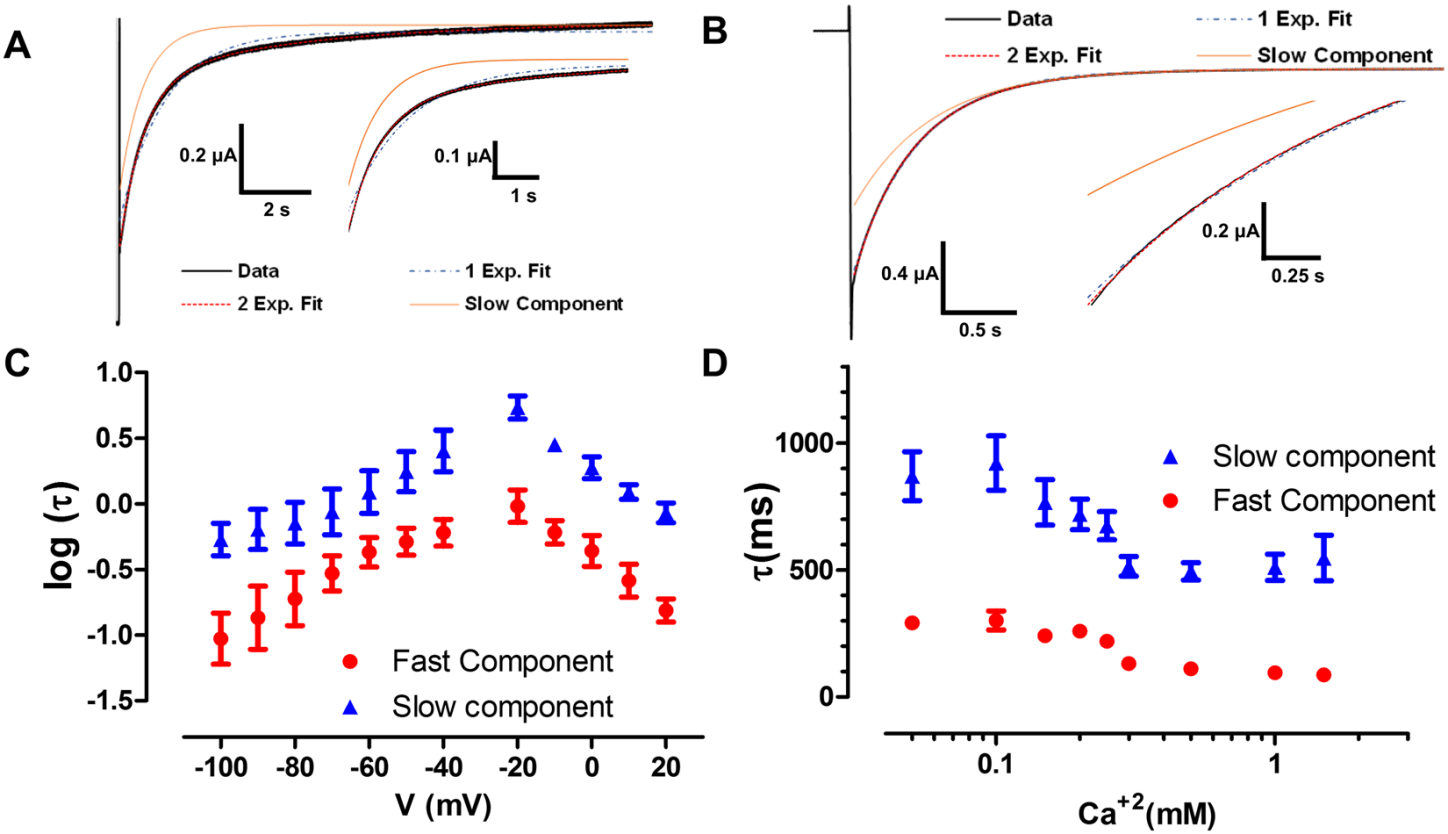
Current relaxation of Cx46 shows two-time constants. (**A)** Activation kinetics seen after a voltage pulse to 0 mV from a holding of −40 mV shows a current that is well fitted by a sum of two exponentials. **(B)** The same is observed in deactivation kinetics when a pulse to −80 mV is given from a holding of 0 mV. The insets an A and B show a magnification of the activation and deactivation current trace to better appreciate the difference between fitting one or two exponentials, this difference is statistically significant. **(C)** At nominal Ca^2+^ concentration, the time constants present intrinsic voltage dependence. As can be seen in **(D)**, Ca^2+^ accelerates the time constants until they reach saturation at high Ca^2+^ concentrations (at −100 mV).

The previous model predicts one voltage-dependent time constant, and at negative voltages this time constant will not be affected by the Ca^2+^ concentration (See Appendix 1). In fact, the deactivation and activation kinetics of the channel does not show a single exponential behavior they are better fitted by the sum of two exponentials (Fig. 4A and B). Therefore, the linear Hill model does not explain the gating kinetics of the Cx46 hemichannel. We measured the activation and deactivation time constants in a voltage range from −100 to 20 mV (Fig. 4C). Both time constants show clear voltage dependence, with a typical inverted U shape. This indicates that there are at least two voltage-dependent transitions. We then measured the Ca^2+^ dependence of the kinetics at −100 mV (Fig. 4D). As can be seen in the graph, both time constants were modified by Ca^2+^ and they saturate at low and high Ca^2+^ concentrations. The presence of two-time constants indicates that there are two rate-limiting steps. The voltage dependence indicates that these rates are intrinsically voltage dependent. On the other hand, if this time constants were given by the rates of Ca^+2^ binding and unbinding one would expect that the time constants tend to zero at high Ca^+2^ concentration. The changes on affinity with voltage and the effect of Ca^2+^ on the time constants indicates that Ca^2+^ binding is somehow coupled to the voltage sensor in an allosteric manner.

**Figure 5:**
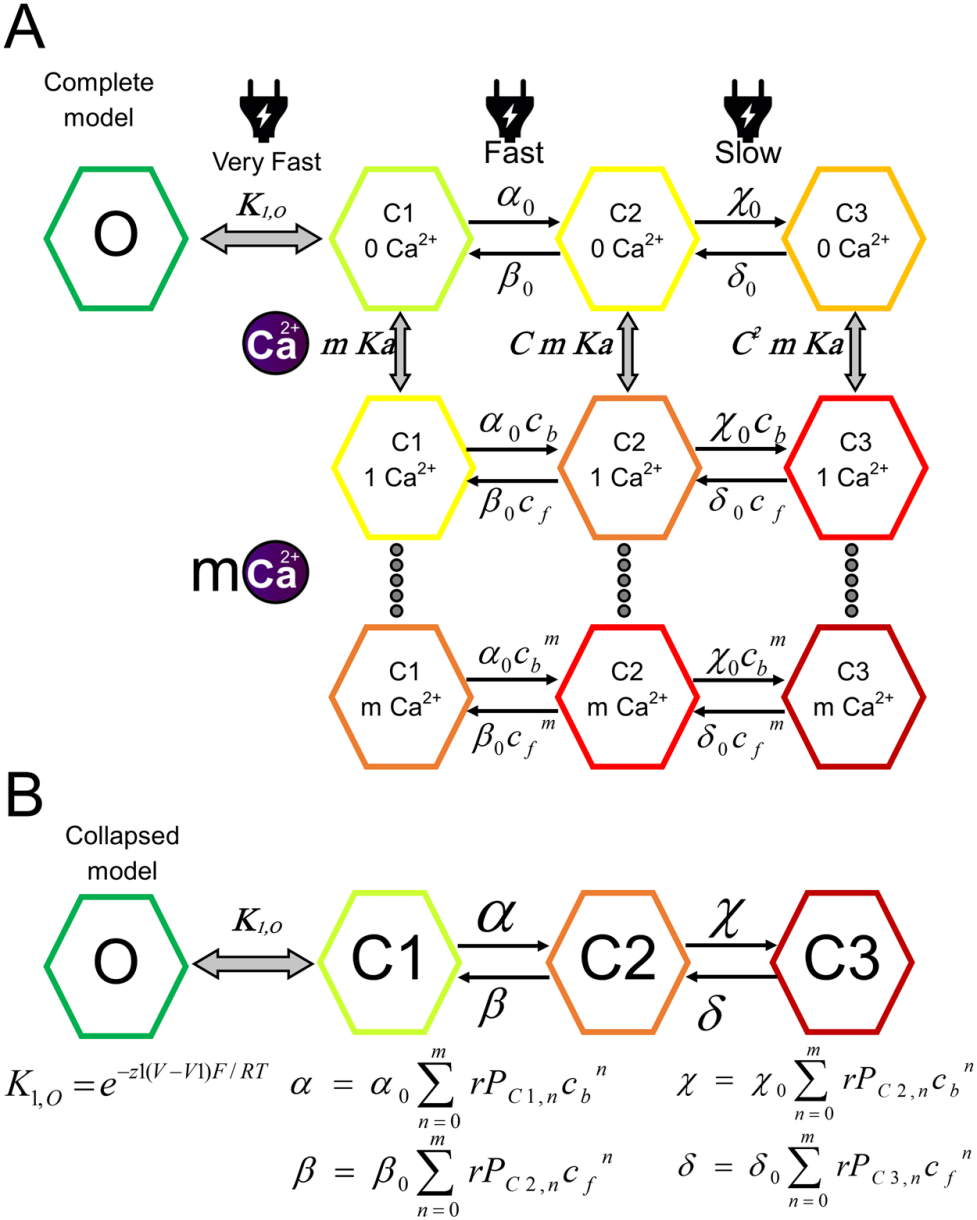
The proposed kinetic mechanism for Ca^2+^ and voltage regulation of Cx46 hemichannels. (**A)** The complete model considers three voltage-dependent transitions form *O* to *C1* to *C2* and *C3*, given by the equilibrium constants K1 and the rates α, β, χ and δ. Binding of Ca^2+^ occurs only in the closed states and is given by the association constant *Ka* (1/*Kd*), m indicates the number of binding sites. Voltage gating and Ca^2+^ binding are allosterically coupled by the coupling constant *C* and its kinetic factors *c*_*b*_ and *c*_*f*_. The black arrows indicate rate-limiting steps and gray arrows indicate transitions in equilibrium.**(B)** The complete model can be collapsed into a simpler model in which each state contains several substates that are in equilibrium. The equations that govern this model are presented.

An allosteric model was developed (Fig. 5A) in which a number *m* of Ca^2+^ binds to the closed states of the channel and promote the voltage dependent transitions to a deeper closed state. Since the Ca^2+^ binding sites are exposed to the extracellular media^6,18^ and the residues described to be involved in Ca^+2^ are in the extracellular loops or the pore^7,15,16,22–24^, we assumed that the Ca^2+^ binding rates are limited by diffusion and thus in equilibrium with respect to the voltage-dependent transitions. This is in accordance with binding kinetics in the orders of milliseconds for many Ca^2+^ binding proteins^25–27^. Since we discarded a binding to the open state and both time constants are affected by Ca^2+^ we expect that the time constants observed reveal transitions between different states of the closed conformation. Thus, our model considers one open state and three closed states to which the *m* Ca^+2^ bind. The effect of voltage and Ca^2+^ indicate that these transitions are voltage dependent and that Ca^2+^ acts allosterically on them. The first open-close transition should be a fast transition in equilibrium with the others, and this step must be voltage dependent, otherwise, we would expect saturation of the inhibition curves at high voltages. Assuming a fast open-closed transition and of diffusion-limited Ca^+2^ binding, allow us to collapse our model into a four-state model (Fig. 5B). This four-state model explains the kinetics observed and all the channel behavior.

### Fitting of the model

**Figure 6:**
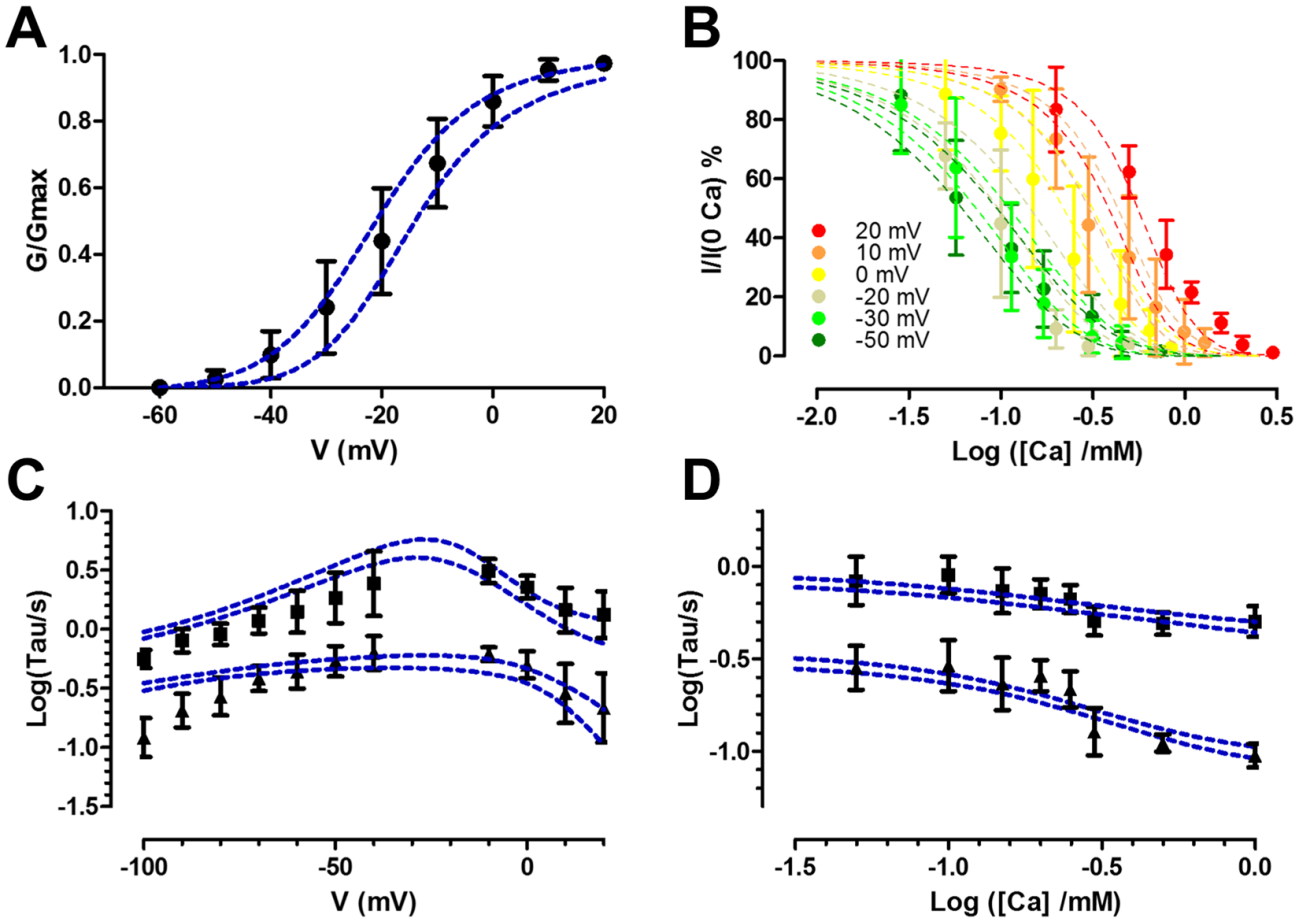
Global fit of the data to the allosteric model with 6 Ca^2+^ binding sites. (**A)** G/Gmax vs voltage. **(B)** Ca^2+^ inhibition curves at different voltages. **(C**) Time constants vs voltage from activation and deactivation kinetics. (**D**) Time constants vs Ca^2+^ concentration from deactivation kinetics at −100 mV. Data is presented as mean ± SD. The lines of the fitting indicate the 95% confidence bands. To display the fitting, we draw 400 sets of parameters from the posterior distribution and the experimental results were simulated based on the proposed model with each parameter set.

To obtain the parameters of the model we performed a global fit of the data. This was based on a maximum likelihood determination and Markov Chain Monte Carlo simulations (MCMC). Bayesian MCMC methods allow to determine parameter identifiability and errors^28^ and to compare different models (For more details see Appendix 2). Since Cx hemichannels are hexamers we tested alternative models with 2, 3 and 6 binding sites, according to the symmetry of the channel. Based on the *odds ratio* (Table 3), the model that best fits the experimental results considers six Ca^2+^ binding sites. In this model, parameters show a unimodal distribution, indicating that they are well constrained by the data (Supplementary Figure 2). Three chains with different starting values were simulated to test convergence, after the burn in period the three chains have converged to the same posterior distribution. (Supplementary Figure 3 **)**. The correlation between parameters is usually low indicating a good exploration of the parameter space by the chain (Supplementary Table 1). From the parameters distributions, we calculated the most likely values and the 95% credible intervals (Table 4). Credible intervals are Bayesian homologs of confidence intervals. The fitting of the model accounts reasonable well for the experimental results (Fig. 6).

**Table 3:** Model comparison: Calculated Odds ratio for models with 2, 3 and 6 Ca^2+^ binding sites against the most likely model (6 sites).

Model	Odds ratio
2 binding sites	5.78 * 10^26^
3 binding sites	1.17 * 10^24^
6 binding sites	1

**Table 4:** Parameters estimates: Most likely values (MLV) and the lower and upper bounds of the 95% credible interval (95% CI) for each parameter of the six binding sites model.

**Parameter**	**MLV**	**95% CI**	
za	0.001	5 * 10^−6^	0.072
zb	0.22	0.13	0.31
zc	0.18	0.12	0.25
zd	1.99	1.66	2.44
A0 (s^−1^)	7.12	5.23	8.14
B0 (s^−1^)	0.92	0.77	1.18
C0 (s^−1^)	0.74	0.57	0.95
D0 (s^−1^)	1.22	0.91	1.52
z1	0.85	0.69	0.96
V1(mV)	−126	−141	−113
Kd(mM)	0.96	0.85	1.13
c_b_	1.11	1.07	1.15
c_f_	0.87	0.76	1.00
C	1.28	1.11	1.46

### Calcium inhibits the flux of water through Cx46 hemichannels

**Figure 7:**
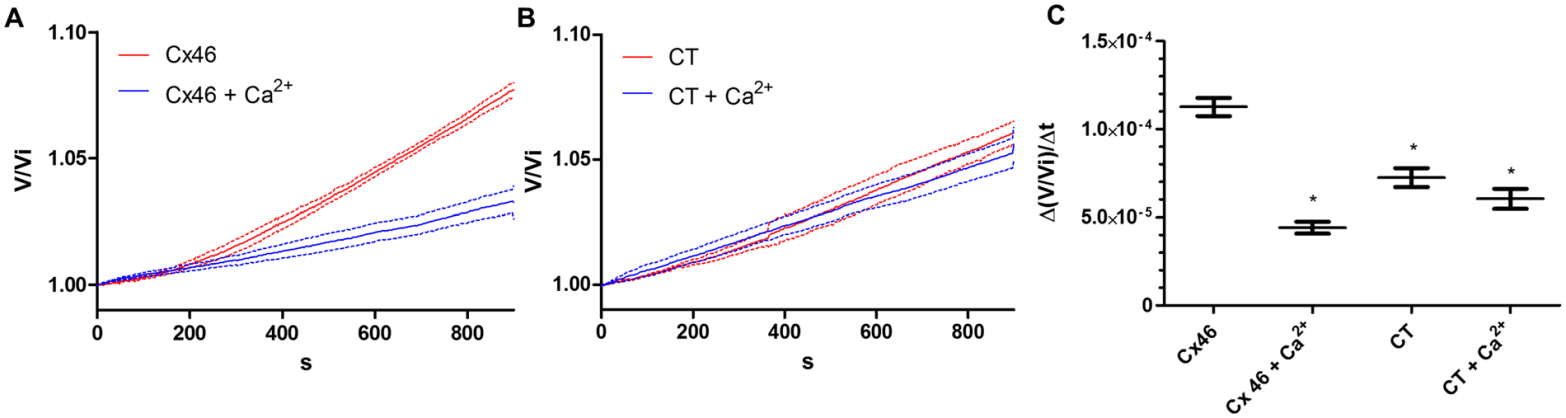
Calcium effects of the flux of water through Cx46 hemichannels. (**A**) Cx46 expressing oocytes were placed in a hypotonic solution with 5 mM Ca^2+^ (Cx46 + Ca^2+^, blue line) or without Ca^2+^ (Cx46, red line). (**B**) Oocytes injected with Cx38 antisense oligonucleotide in a hypotonic solution with 5 mM Ca^2+^ (CT + Ca^2+^, blue line) or without Ca^2+^ (CT46, red line). The relative volume of oocytes vs. time was plotted for both conditions; the results are shown as mean (continuous line) ± SEM (dashed lines). (**C**) The rate of volume change was calculated for the last 50 s of the recordings. The Cx46 condition shows a significantly higher rate of volume change than the Cx46 + Ca^2+^ condition and with control oocytes in the presence or absence of Ca^2+^. *p < 0.05 in comparison to Cx46 using Student’s t-test (n = 4 in all experimental conditions).

Our results clearly agree with the stabilization of the closed state by Ca^2+^ and not with the electrostatic barrier mechanism. To experimentally test whether Ca^2+^ binding stabilizes a conformation in which the Cx46 pore has contracted, we measured volume changes of oocytes injected with Cx46 in the presence or absence of Ca^2+^ under hypotonic conditions. If Ca^2+^ is stabilizing a closed configuration of the Cx46 hemichannel, we expect to detect a clear decrease of the water flow in the presence of the Ca^2+^. On the other hand, a purely electrostatic mechanism (an electrostatic seal as proposed by Bennet *et al*.^15^), being water a dipole, should not hinder water flow through the channel. Oocytes were placed in a hypotonic solution and the volume of oocytes was tracked. Volume relative to the initial volume (V/Vi) was calculated. After 900 s oocytes expressing Cx46 showed an increase in the volume of about 8% in conditions without Ca^2+^, in 5 mM of Ca^2+^ the volume change was about 3% (Fig. 7A). In control oocytes, a volume change around 5% was still observed, but this change is insensitive to external Ca^2+^ (Fig. 7B). The rate of volume changes is lower in the Ca^2+^ added condition than in the Ca^2+^-free conditions, and shows no difference with oocytes injected with Cx38 antisense only (CT) (Fig. 7C).

## Discussion

In this study, we address the interplay between Ca^2+^ and voltage in the regulation of Cx46 hemichannels. Since both regulatory mechanisms are ubiquitous among Cx hemichannels we expect that our findings are pertinent to other Cx isoforms. The present study supports the idea that Ca^2+^ and voltage act synergistically on the slow gate. Under physiological conditions, the extracellular concentration of Ca^2+^ and typical membrane voltage of a resting cell would maintain most Cx hemichannels closed. Nevertheless, conditions that lower local extracellular Ca^2+^ concentration such as repetitive activity in neurons^29,30^ may promote the opening of hemichannels even at resting potentials.

It was initially proposed by Ebihara *et al*. that Ca^2+^ inhibited Cx hemichannels through a voltage-dependent block, followed by a voltage-dependent binding to this blocked state^6^. The voltage-dependent stabilization of the closed state comes from an experiment where the divalent cation was added while keeping the channel closed, after the application a reduction of the current induced by depolarization was observed^6^. This experiment argues in favor of a stabilization of the closed state (or blocked state as defined in this first model). The voltage-dependent block was called into question by Verselis *et al*., who showed that Cx46 hemichannels did close even in the absence of divalent cations, and adding divalent cations produced longer-lived closed states^18^. These results indicate that divalent cations stabilize the closed state of the channel but whether this binding is voltage dependent is still unknown. Also, they do not discard the binding of Ca^2+^ to the open state. Moreover, a new structure of the gap junctional channel by Bennet *et al*. showed binding of Ca^2+^ to the open conformation, to which they propose an electrostatic barrier as the mechanism through which Ca^2+^ might affect the permeation^15^. We note here that this electrostatic mechanism is mechanistically equivalent to a block of the open state and is at odds with Verselis *et al*. results^18^.

In this work, we addressed the following issues: Can Ca^2+^ bind to the open state? Is the Ca^2+^ binding voltage dependent? What is the mechanism through which Ca^2+^ may be stabilizing the closed states? Considering the new evidence we gathered here, we also re-examined the electrostatic barrier or closed state stabilization mechanism for Ca^2+^ inhibition. We relied on the ability of voltage to modify the open-closed equilibrium. We found that as we lower the voltage of the test pulse the apparent affinity of Ca^2+^ increases and saturates at negative potentials (−30 and −50 mV). Altogether the present results can only be explained by a voltage-independent stabilization of the closed state of the channel by Ca^2+^. A possible interference in our results can come from the presence of Mg^2+^ in the recording solution. The presence of Mg^2+^ is necessary to avoid endogenous currents that arise in the absence of divalent cations. The effect of Mg^2+^ in the channel is similar to Ca^2+^ but with a lower affinity^18^, so it is expected that Mg^2+^ will be displaced by Ca^2+^ in our experiments, especially at high Ca^2+^ concentrations.

We tested several linear binding models to describe Ca^2+^ binding to Cx46. The best model that accounts for Ca^2+^ affinity utilizes the Hill equation as an approximation for binding in the closed state. It has been shown that even for simple models with two binding sites it is not possible to obtain the parameters of the model from binding curves alone^28^. Thus, even though the Hill equation has no clear physical meaning due to the assumption of infinite cooperativity, it is useful to quantify Ca^2+^ affinity of the Cx46 hemichannel.

To test the suitability of the linear model, we measured the voltage and Ca^2+^ dependence of the channel kinetics. From a linear model with only one voltage-dependent transition, we would expect that the kinetics show only one exponential in absence Ca^2+^. It is also expected that Ca^2+^ should not affect the kinetics at very negative potentials. In fact, Cx46 hemichannels kinetics are better explained by two exponentials, and both components are voltage-dependent. This has also been observed by other groups^14^. The kinetics at −100 mV also showed Ca^2+^ dependence, with saturation at high Ca^2+^ concentration. We could explain the voltage-dependent time constants using a linear model with two voltage-dependent steps, but the Ca^2+^ dependence of the deactivation should not be affected in this kind of model. If the time constants were determined by the rates of Ca^2+^ binding and unbinding we expect them to tend to zero at high Ca^2+^ concentration, which they don’t. Thus, we are certain that voltage and Ca^+2^ dependence of Cx46 cannot be explained by linear models, but a model in which Ca^2+^ binding and voltage sensing are allosterically coupled.

An allosteric model is able to account for all the observed phenomena; the saturation of the IC50 at negative potentials, the decrease in the apparent affinity with higher voltages and the Ca^2+^ and voltage dependence of the channel kinetics. In this model, the first transition is voltage dependent and not affected by Ca^2+^. The existence of this transition is important to explain the change in Ca^2+^ affinity with voltage. If this transition is not voltage-dependent one would expect a saturation of the inhibition curve at high potentials. We assume that the kinetics of this first transition is too fast, probably in the order of milliseconds, to be observed under our experimental conditions (the voltage electrode can take up to 5 ms to reach the command value). The binding of Ca^2+^ is considered to be limited by diffusion and thus in equilibrium with respect to the transition between the voltage-dependent steps. This consideration allows us to collapse a model with 10 or more states into four states with rates depending on the relative occupation of all the states in the model given in Fig. 5A. This reduced model has only two voltage- and Ca^2+^-dependent time constants and is the most parsimonious model able to account for the equilibrium and kinetic data presented in this study. It is possible that other models, such as binding to intermediate states could generate the same effects as the one observed, but we discard these models because they do not generate inhibition curves that saturates at negative voltages.

What can we learn from the allosteric model? The model shows that Ca^2+^ binding and voltage sensor movement are coupled. This coupling is reflected by the coupling constant C which is given by c_b_/c_f_ and is 1.28 (see Table 4). This means that the Ca^2+^ binding constant that defines the *C3-C3*Ca equilibrium is around 1.6 times larger than the one that defines the *C1-C1*Ca equilibrium. In this model, Ca^2+^ binding also facilitates transitions between closed states. The factors *c*_*b*_ and *c*_*f*_ reflect the effects of Ca^2+^ in the backward and forward rates, which are 1.1 and 0.87 respectively (see Table 4). Even though these values seem to be small we have to consider that there are six binding sites and each one is affecting the voltage sensor and vice versa. These values explain the acceleration of the kinetics observed in the deactivation currents and the slowing down of the activation currents with Ca^2+^. The model also shows that the voltage-dependence of the deactivation rates is small compared to that of the activation rates. This is similar to previous observations in Cx26 hemichannels and might be a characteristic among connexins^16,31^. Three voltage-dependent transitions in a hexamer, are consistent with a concerted movement of two adjacent subunits that give rise to each voltage-dependent step. The model additionally predicts that the mean open time is independent of Ca^+2^ and that a fast-kinetic step that reflects the *O* to *C1* transition is expected.

Verselis *et al*. observed that in the absence of divalent cations single channel recordings of Cx46 hemichannels show very brief closures and that extreme negative voltages are needed to close the channels (−160 mV)^18^. These observations are in reasonable agreement with the assumption that the open-closed transition is in equilibrium with respect to deeper closed transitions.

In Cx26 the deactivation time constants show a single exponential, voltage and Ca^2+^ dependence^16,18^. Lopez *et al*. interpret their data based on a two-states kinetic model in which Ca^2+^ binding destabilizes the open state, which is equivalent to a Ca^2+^-blocking model. However, the evidence against a Ca^2+^ block in Cxs provided by Verselis and the fact that the D50N mutation impairs Ca^+2^ inhibition without a strong effect on the voltage-dependence of the channel make this mechanism untenable^16,18,24^. In a block model, the *Kd* can be obtained from the inhibition or from the time constant vs Ca^2+^. Lopez *et al*. found in Cx26 that the *Kd* obtained from inhibition and kinetics are 0.33 and 0.72 respectively and they interpreted the difference due to effects of Ca^+2^ on the open and closed state^16^. Using the allosteric model, we do not need to appeal to these differential effects. The difference between the *Kd* obtained between the inhibition curves and the kinetics can be reproduced in the allosteric model with a large backward coupling constant *c*_*b*_ and a low *Kd*. We can also explain the single exponential behavior allowing to the transition between the *C1* and *C2* state to be the slowest rate of the system, this will mask the kinetic effects of the deeper transitions. The application of the allosteric model to Cx26 can help to explain how the D50N mutation has a strong effect on Ca^+2^ inhibition without impairing the voltage dependence^16,24^. As Sanchez *et al*. noticed, it is probable that D50N mutation uncouples Ca^2+^ binding from loop gating^24^.

The recent Ca^2+^ bound structure of Cx26^15^ showed that Ca^2+^ binds to pore-lining residues that are part of the proposed gate and voltage sensor^31–33^. The structure obtained does not show any important structural differences between the Ca^2+^ bound and unbound structures. To explain this finding, Bennet *et al*.^15^ suggested that bound Ca^2+^ acts as the electrostatic barrier preventing cation flux. It is possible that the constrain imposed by the formation of the gap junction in the extracellular loops might prevent the conformational changes and formation of the Ca^2+^ binding sites present in hemichannels. It is possible that Ca^2+^ can affect the channel conductance through several mechanisms, regulation of the open-closed equilibrium and direct regulation of ion permeation both mechanisms having differential roles in the regulation of hemichannels and gap junction channels. Our results are consistent with those of Lopez *et al*. in which they show that modification of glycine-45 in Cx26 when mutated to cysteine (G45C), can be modified by the negatively charged MTSES and Cd^2+^, and that this modification is not affected by the presence of Ca^2+^ ^22^. The G45C mutant is inhibited by Ca^2+^ indicating that the presence of Ca^2+^ does not generate an electrostatic barrier near the glycine-45 residue in Cx26 hemichannels. In line with this, we performed water permeability assays in oocytes expressing Cx46. When Ca^2+^ was added to the bath, the level of water influx into the oocyte was decreased. If the electrostatic plug is present, water being a dipole should not be as much affected as ions in its flux through hemichannels. Therefore, our results indicate that the presence of Ca^2+^ promotes the closure of the pore to levels that significantly hinder the flux of water.

The proposed model has direct mechanistic implications since it implies that part of the voltage sensor of the slow gate must move before Ca^2+^ can inhibit the channel. By stabilizing the closed state of Cxs channels, Ca^2+^ acts as a safety valve, keeping hemichannels closed and avoiding the deleterious consequences of their opening. The simplest mechanism we can think of is one in which negative charges in the voltage sensor/pore of Cx hemichannels are responsible for Ca^2+^ binding but they are only reachable by Ca^2+^ when voltage sensors acquired their resting conformation. One can think of Ca^2+^ as a “foot in the door” but unlike the block of Kv channels by tetraethylammonium^34^, it would “immobilize” the gating charge in its “resting” conformation. Finally, we believe that the allosteric model presented in this work will be of importance as a conceptual framework to understand Cxs hemichannel gating.

## Materials and Methods

### *In vitro* transcription and expression in *Xenopus laevis* oocytes

Rat Cx46 pSP64T was kindly provided by Dr. Lisa Ebihara, Rosalind Franklin University. Cx46 DNA was linearized using the EcoRI enzyme (Fermentas) and transcribed using the *in vitro* transcription kit SP6 mMessagemMachine systems (Ambion) following manufacturer’s instructions. Stage V and VI oocytes were selected, defolliculated and injected with 5 ng of the RNA plus Cx38 antisense to null endogenous expression of Cx38^35^. Oocytes were incubated at 18 °C in ND96 medium containing (in mM): 96 NaCl, 1 KCl, 1 MgCl_2_, 10 HEPES and 1.8 CaCl_2_. Experiments were done 24–48 hrs post injection.

### Electrophysiology

Oocytes were injected with 0.25 nmol of BAPTA before the experiments. Hemichannel currents were obtained and using the two-electrode voltage clamp technique. Currents were acquired and filtered to 1 kHz with an “Oocyte clamp” (Warner Instruments). Electrodes were filled with a 3 M KCl solution and exhibited resistances between 0.5 and 2 MΩ. The bath solution contained (in mM): 88 choline chloride, 1 KCl, 1 MgCl_2_ and 10 HEPES adjusted to pH 7.4. Macroscopic currents were sampled at 1 kHz using an analog to digital converter “Digidata 1200” (Axon instruments). Ca^2+^ was added to the recording chamber to obtain the desired concentration.

### Data analysis and equations

The *I*_*tail*_ *(V)/I*_max_ –voltage data was fitted using a Boltzmann function:

1 $$\frac{{I}_{tail}(V)}{{I}_{\max }}=\frac{1}{1+{e}^{-z\delta (V-{V}_{1/2})F/RT}}$$

where *I*_*tail*_(V) is the tail current at voltage *V*, *I*_*max*_ is the maximum tail current, *zδ* is the apparent number of gating charges *z* multiplied by a fraction of the field *δ* they cross, *V*_*1/2*_ the voltage at which the tail current is half maximal. *R, T*, and *F* have their usual meanings.

The Ca^2+^ inhibition curves were fitted using the Hill equation:

2 $$I(Ca)/I(0Ca)=\frac{1}{1+{(\frac{[C{a}^{+2}]}{IC50})}^{n}}$$

where *I(Ca)* is the tail current at a given Ca^2+^ concentration, *I(0Ca)* is the tail current without Ca, [Ca^2+^] is the Ca^2+^ concentration and *n* the Hill slope. The actual values used for the fitting are the Log [Ca^2+^] and Log IC50. It must be noted that the Hill slope represents the number of binding sites only in the case of infinite cooperativity between the binding sites. Nevertheless, it is relevant to the phenomenological description of the inhibition curves.

For the modeling of Cx46 inhibition by Ca^2+^ we used several linear and nonlinear models which are explained in detail in Appendix 1.

Graph figures and fittings of Figs 1, 2, and Supplementary 1 were done with Prism (GraphPad). The optimization of parameters was performed by minimizing squared errors. For the fitting of all the inhibition curves to a single model and calculate the parameters errors we used NonLinearModelFit algorithm built in the Mathematica software.

### The allosteric model

The model presented in Fig. 5 assumes that in the absence of Ca^2+^ all transitions from *O* to *C3* are voltage dependent and controlled by the slow gate. If we assume that the binding of Ca^2+^ and the first open-closed transition are in equilibrium with respect to the movement of the voltage sensors in the other closed states, this model can be collapsed into a four-states model, each one comprising several substates. The first state is called *O* and considers only the open state, this is in equilibrium with the *C1* unbounded state. The *C1* state comprises the Ca^2+^ bounded and unbounded *C1*_*n*_ state, where *n* denotes the number of Ca^2+^ bounds. The states *C2* and *C3* comprise Ca^2+^ bounded and unbounded states of *C2*_*n*_ and *C3*_*n*_. This model is treated in depth in Appendix 1.

### Markov Chain Monte Carlo (MCMC)

The global fitting of all the experimental data to a single mechanistic model with several calcium-binding sites and several voltage-dependent steps was done using a custom-made MCMC script in Mathematica (Wolfram 10.3). MCMC methods are a class of algorithms that allows us the sampling from a distribution based on constructing a Markov chain that has the desired distribution as its equilibrium distribution. Among the advantages of this methodology is the ability to assess parameter identifiability and calculation of parameter errors in complex models such as the one presented here^28,36^.

This methodology is based on the Bayes’ theorem, which for the parameters of a model can be written as:

3 $$P(\theta /D)=\frac{P(\theta )P(D/\theta )}{P(D)}=\frac{P(\theta )P(D/\theta )}{\int P(\theta )P(D/\theta )d\theta }$$

where *P(θ/D)*represents the probability or probability density function of a set of parameters θ given the new information (the experimental data) also called the posterior probability, *P(θ)* is the prior probability of the parameters (before considering the experimental data), *P(D/θ)* is the likelihood of the data given the set of parameters *θ* and *P(D)* is the probability of the data itself. It can be though more empirically as an integration constant that makes the integral of *P(θ/D)* over all the parameter space equal to one. A detailed explanation of the approach utilized can be found in Appendix 2.

### Water permeability assay in *X. laevis* oocytes

Analysis of volume changes of oocytes was based on the methodology described for aquaporin water channels^37,38^. Oocytes injected with Cx46 and Cx38 antisense or Cx38 antisense only (CT) were left in a hypotonic solution containing (in mM) 44 NaCl, 0.5 KCl, 0.5 MgCl_2_ and 5 HEPES adjusted to pH 7.4, 5 mM CaCl_2_ was added to promote hemichannel closing. Videos of the oocytes were acquired using a Logitech® Webcam C920 HD Pro mounted on an Olympus BX51WI microscope with a 5X objective using Oasis software version 1.00 (Oasis Scientific). Each frame was converted to images using Virtual Dub software version 1.10.4. The images were analyzed in Mathematica 10.3 (Wolfram) using a custom made script. The script calculated the volume of the oocyte based on the area at the focal plane and assuming spherical symmetry. Relative volume (*V/V*_*i*_) was calculated in 1 frame for each second. The rate of volume change was calculated using the last 50 seconds of the recording.

### Data availability

The datasets generated during and analyzed during the current study are available from the corresponding author on reasonable request.

## Electronic supplementary material

Supplementary Information
