# Supplementary material for: Calcium binding and voltage gating in Cx46 hemichannels
Source: Sci Rep. 2017 Nov 20;7:15851. doi: 10.1038/s41598-017-15975-5 (PMC5696461; doi:10.1038/s41598-017-15975-5)
Supplement: Supplementary file 1 — Supplementary Information [file 41598_2017_15975_MOESM1_ESM.doc]

**Calcium binding and voltage gating in Cx46 hemichannels**.

Bernardo I. Pinto, Amaury Pupo, Isaac E. García, Karel Mena-Ulecia, Agustín D. Martínez, Ramón Latorre^*^ and Carlos González^*^

Appendix 1: Modeling of the Cx hemichannel

**Inhibition curves predicted by different models considering different modes of Ca^2+^ binding to the hemichannel**

The Ca^2+^ inhibition curves derived from several models were tested in their capability to reproduce the observed effects of Ca^2+^. We define first the Ca^2+^ dissociation constants (*Kd_i_*), the equilibrium constants (*Ki*) and the voltage dependent constant (*K_V_*) as:

$Kd_{i}=\frac{CCa_{i-1}[Ca^{+2}]}{CCa_{i}}$ (s1)

$Ki=\frac{CCa_{i-1}}{CCa_{i}}=\frac{Kd_{i}}{[Ca^{+2}]}$ (s2)

$K_{V}=e^{z\delta(V-V_{1/2})F/RT}$ (s3)

where *CCa_i_* and *C* number of channels in the closed with *i* Ca^2+^ bounds and no Ca^2+^ bound closed states, respectively. Dissociation constants *Kd_i_* are related to equilibrium constants *Ki* by equation (s2).

1. *Sequential model with n Ca^2+^ -bound closed states*

In a linear model with *n* Ca^2+^ bound states, where only the open-close transition is voltage-dependent, the open probability (*P_O_*) is defined as:

$\begin{matrix} P_{O}=\frac{O}{O+C+CCa_{1}\bullet\bullet\bullet+CCa_{n}} \\ =\frac{KnK(n-1)\bullet\bullet\bullet K1K_{V}}{1+Kn+(Kn*K(n-1))\bullet\bullet\bullet+(KnK(n-1)\bullet\bullet\bullet K1K_{V})} \end{matrix}$ (s4)

At 0 Ca^2+^ concentration eq, (s4) becomes:

$P_{O[0Ca]}=\frac{K_{V}}{1+K_{V}}$ (s5)

And the ratio between the open probabilities of the 0 Ca^2+^ condition (*P_O[0 Ca]_*) and the different Ca^2+^ concentrations (*P_O[Ca]_*) is:

$\begin{matrix} \frac{P_{O[Ca]}}{P_{O[0Ca]}}=\frac{KnK(n-1)\bullet\bullet\bullet K1K_{V}}{1+Kn+(KnK(n-1))\bullet\bullet\bullet+(KnK(n-1)\bullet\bullet\bullet K1K_{V})}\frac{1+K_{V}}{K_{V}} \\ =\frac{KnK(n-1)\bullet\bullet\bullet K1(1+K_{V})}{1+Kn+(KnK(n-1))\bullet\bullet\bullet+(KnK(n-1)\bullet\bullet\bullet K1K_{V})} \end{matrix}$ (s6)

Given the present data, a more compact model that treats the binding of Ca^2+^ as a Hill Model is preferable over the sequential binding models. In this linear Hill model the *Kd* and *K1* constant become

$Kd=\frac{C[Ca^{+2}]^{n}}{CCa_{n}}$ (s7)

$K1=\frac{C}{CCa_{n}}=\frac{Kd}{[Ca^{+2}]^{n}}$ (s8)

In this model, the *P_O_* is defined by:

$P_{O}=\frac{O}{O+C+CCa_{n}}=\frac{K1K_{V}}{1+K1+K1K_{V}}$ (s9)

And the ratio between the open probabilities of the 0 Ca^2+^ condition and the different Ca^2+^ concentrations is:

$\frac{P_{O[Ca]}}{P_{O[0Ca]}}=\frac{K1K_{V}}{1+K1+K1K_{V}}\frac{1+K_{V}}{K_{V}}=\frac{K1(1+K_{V})}{1+K1(1+K_{V})}=\frac{1}{1+\frac{1}{K1(1+K_{V})}}$ (s10)

Therefore, *IC50* at different voltages is given by:

$\frac{P_{O[Ca]}}{P_{O[0Ca]}}=\frac{1}{2}=\frac{1}{1+\frac{1}{K1(1+K_{V})}}$ (s11)

Combining eqs. (s7) and (s11), we have

$\frac{1}{1+K_{V}}=K1=\frac{Kd}{IC50^{n}}$ (s12)

Using eqs. (s3) and (s12):

(s13)

At large negative voltages *K_V_* <<1 and,

(s14)

1. *Voltage-dependent Ca^2+^ binding to the closed state:*

In the case of a voltage-dependent Ca^2+^ binding (**Suppl. Figure 1A**), the *K*1 constant is now multiplied by a voltage-dependent term:

$K1(V)=\frac{Kd}{[Ca^{+2}]^{n}}e^{-2n\delta CaVF/RT}$ (s15)

where δCa represents the fraction of the electric field that each Ca^2+^ ion traverses.

In this model the IC50 becomes:

(s16)

Applying logarithm to both sides of equation s16 we get:

$\log(IC50)=\frac{1}{n}\log\left(Kd\left(e^{-2n\delta CaVF/RT}\right)\left(1+e^{z\delta(V-V_{1/2})F/RT}\right)\right)$ (s17)

At large negative voltages equation, s17 becomes:

$\log(IC50)=\frac{1}{n}\log\left(Kd\left(e^{-2n\delta CaVF/RT}\right)\right)$ (s18)

Equations s17 and s18 show that the Log *IC50* does not saturate at negatives voltages, which is inconsistent with the equilibrium results of Fig. 2D (**Supp. Figure 1E, red solid line))**.

.

1. *Preferential Ca^2+^ binding to the closed state*

In a model in which Ca^2+^ binds to the open state with less affinity than to the closed state (**Suppl. Figure1B**), the *P_O_* is defined as:

$P_{O}=\frac{O+OCa_{n}}{O+C+CCa_{n}+OCa_{n}}=\frac{rK1K_{V}+rK_{V}}{1+rK1+rK_{V}+rK1K_{V}}$(s19)

Where *r* represents the ratio between the *Kd* of the closed and open states.

Since in this case saturating Ca^2+^ concentrations might not generate a complete inhibition of current (**Suppl. Figure 1B**), we use an alternative expression for the *IC50*:

$\frac{(I(Ca)-I(sCa))}{(I(0Ca)-I(sCa))}=\frac{1}{2}=\frac{1}{1+\left(\frac{[Ca^{+2}]}{IC50}\right)^{n}}$ (s20)

Where *I(sCa)* represents the current at saturating Ca^2+^ concentrations.

In this case, the IC50 can be expressed as:

(s21)

Applying logarithm to both sides of equation s21 we get:

$\log(IC50)=\frac{1}{n}\log\left(\frac{Kd\left(r+re^{z\delta(V-V_{1/2})F/RT}\right)}{1+re^{z\delta(V-V_{1/2})F/RT}}\right)$ (s22)

At high voltages equation, s22 becomes:

$\log(IC50)=\frac{1}{n}\log\left(Kd\right)$ (s23)

And at large negative voltages equation s24 becomes:

$\log(IC50)=\frac{1}{n}\log\left(rKd\right)$ (s24)

Thus the Ca^2+^ inhibition curves in this model saturate at large negative and at high voltages (**Supp. Figure 1E, blue solid line)**.

1. *Specific Ca^2+^ binding to the open state*

If Ca^2+^ binds only to the open state of the channel and this binding generates a new closed state independent form the intrinsic voltage gating mechanism (**Suppl. Figure 1C**), this can be modeled as an open state block. In this model, the *P_O_* is defined as:

$P_{O}=\frac{O}{O+C+CCa_{n}}=\frac{K_{V}}{1+K_{V}+\frac{K_{V}}{K1}}$ (s25)

In this case, CCa_n_ represent the number of channels in the “*blocked*” state.

In this case, the IC50 can be expressed as:

(s26)

Applying logarithm to both sides of equation s26 we get:

$\log(IC50)=\frac{1}{n}\log\left(\frac{Kd\left(1+e^{z\delta(V-V_{1/2})F/RT}\right)}{e^{z\delta(V-V_{1/2})F/RT}}\right)$ (s27)

At high voltages equation, s27 becomes:

$\log(IC50)=\frac{1}{n}\log\left(Kd\right)$ (s28)

We can also deduce from equation s28 that the Log *IC50* grows with decreasing voltages and that this curve does not saturate (**Supp. Figure 1E, black solid line)**.

1. Ca^+2^ binding to intermediate states

It is possible that Ca^2+^ is favoring the movement to deeper closed states in the channel. We can think of two equivalent states that are connected by an intermediate Ca^2+^ binding step (**Suppl. Figure 1D**). To simplify this model, the voltage-dependent states are described using the same equilibrium constant K1.

In this model, the *P_O_* is defined as:

$P_{O}=\frac{O}{O+C1+C1Ca_{n}+C2Ca_{n}}=\frac{Kv^{2}K1}{1+K_{V}+KvK1+Kv^{2}K1}$ (s29)

Here, *C*1, *C*1*Ca_n_* and *C*2*Ca_n_* represent the number of channel in the first closed, first closed and Ca^2+^ bound and second closed state. See model below **Supp. Figure 1D**

In this case, the IC50 can be expressed as:

(s30)

Applying logarithm to both sides of equation s30 we get:

$\log(IC50)=\frac{1}{n}\log\left(Kd\left(e^{z\delta(V-V_{1/2})F/RT}\right)\right)=\frac{z\delta(V-V_{1/2})F}{nRT}*\log(e)+\frac{1}{n}\log\left(Kd\right)$ (s31)

We can see from equation s31 that the Log *IC50* in this model becomes linear with voltage (**Supp. Figure 1E, green solid line)**.

Kinetics of the linear Hill model

The rates in the linear Hill model can be written as:

$$O\leftrightarrow_{k_{ON}}^{k_{OFF}} C\leftrightarrow_{k_{D}}^{k_{A}*Ca^{n}} CCa_{n}$$

The kinetics of a tree states system, as the one presented in the linear Hill model, have two-time constants, these have been previously derived analytically [^1^](#_ENREF_1). The inverse of the time constants for this model would be:

$\frac{1}{\tau}=\frac{k_{OFF}+k_{ON}+k_{D}+k_{A}Ca^{n}}{2}\pm\sqrt{\left(\frac{-k_{OFF}+k_{ON}+k_{D}+k_{A}Ca^{n}}{2}\right)^{2}+(k_{D}-k_{OFF})k_{ON}}$(s32)

Since *k_ON_* and *k_OFF_* are voltage dependent, we expect that at very negative voltages and *k_OFF_* gets larger and *k_ON_* tends to 0. Thus equation (s32) can become:$\frac{1}{\tau}=\frac{k_{OFF}+k_{D}+k_{A}Ca^{n}}{2}\pm\sqrt{\left(\frac{-k_{OFF}+k_{D}+k_{A}Ca^{n}}{2}\right)^{2}}$ (s33)

The solutions of s33 are

$\frac{1}{\tau}=k_{OFF}$ (s34)

$\frac{1}{\tau}=k_{D}+k_{A}Ca^{n}$ (s35)

Thus, the linear Hill model at very negative potentials is expected to show one voltage-dependent and Ca^2+^-independent time constant. If the Ca^2+^ binding step is much faster than the voltage-dependent step this would be the only time constant observed. Otherwise, we expect a second time constant that reflects the Ca^2+^ binding steps. It also has to be noted that the voltage-dependent time constant is equivalent to the mean open time of the channel.

**The allosteric model**

The model presented in Figure 5A assumes that in the absence of Ca^2+^ all transitions from *O* to *C3* are voltage dependent. If we assume that the binding of Ca^2+^ and the first open-closed transition are in equilibrium with respect to the movement of the voltage sensors in the other closed states, this model can be collapsed into a four-state model shown in Figure 5B, each one comprising several substates. The first state is called *O* and considers only the open state, this is in equilibrium with the *C1* unbounded state. The *C1* state comprises the Ca^2+^ bounded and unbounded *C1_n_* state, where *n* denotes the number of Ca^2+^ bound. The states *C2* and *C3* comprise Ca^2+^ bounded and unbounded states of *C2_n_* and *C3_n_*.

In order to define the probabilities of this model we need to define the partition function (***Z***) within each collapsed state, which is given by the dissociation constant and the allosteric coupling factor *C*, and in the case of state *O* by the voltage dependent equilibrium constant between the open and first closed state (*K_1,O_*).

$Z_{O}=1+K_{1,O}$ (s36)

$Z_{C1}=\left(1+\frac{\left[Ca^{2+}\right]}{Kd}\right)^{m}$ (s37)

$Z_{C2}=\left(1+\frac{\left[Ca^{2+}\right]C}{Kd}\right)^{m}$ (s38)

$Z_{C3}=\left(1+\frac{\left[Ca^{2+}\right]C^{2}}{Kd}\right)^{m}$ (s39)

The exponent *m* indicates the number of binding sites of the model and *K_(1,O)_* is given by:

$K_{(1,O)}=e^{-z1(V-V1)F/RT}$ (s40)

where *z1* is the voltage dependence of the transition and *V1* the voltage at which *K_(1,O)_* is 1.

We then can write the relative probability of each substate. For the open probability (*P_O_^rel^*) this will be:

$P_{O}^{rel}=\frac{1}{Z_{O}}$ (s41)

For the substates of the *C1* state this will be:

(s42)

where *P_C1n_^rel^* is the relative probability of the *n*Ca^2+^ bound state within the *C1* state. For the relative probability of the *C2* and *C3* states (*P_C2n_^re^*^l^ and *P_C3n_^rel^*) the expressions are:

(s43)

(s44)

In this collapsed model, the rates describing the transit between states correspond to the sum of the rates of every substate weighted by their relative probability [^2^](#_ENREF_2). We also have to consider that the allosteric coupling constant **C** has to be split to reflect its effect on the rates of the voltage sensor, from the *C3* to *C1* transition (forward, *c_f_*) and from *C1* to *C3* (backwards, *c_b_*). As an example the rate constant from the *C3* state to *C2* state in a model with 2 Ca^2+^ binding sites would be:

$\delta=\delta_{0}P_{C3,0}^{rel}+\delta_{0}rP_{C3,1}^{rel}c_{f}+\delta_{0}rP_{C3,2}^{rel}c_{f}^{2}$ (s45)

where *δ_0_* is the voltage-dependent rate from the unbounded *C3* state to the unbounded *C2* state. This can be expressed more generally for *m* numbers of binding sites as:

$\delta=\delta_{0}\sum_{n=0}^{m} P_{C3,n}^{rel}c_{f}^{n}$ (s46)

Using the same reasoning we can write the equations for the other rates:

$\alpha=\alpha_{0}\sum_{n=0}^{m} P_{C1,n}^{rel}c_{b}^{n}$ (s47)

$\beta=\beta_{0}\sum_{n=0}^{m} P_{C2,n}^{rel}c_{f}^{n}$ (s48)

$\chi=\chi_{0}\sum_{n=0}^{m} P_{C2,n}^{rel}c_{b}^{n}$ (s49)

The voltage-dependent rates are described by the following equations:

$\alpha_{0}=A0e^{-zaFV/RT}$ (s50)

$\beta_{0}=B0*e^{zb*F*V/RT}$ (s51)

$\chi_{0}=C0*e^{-zc*F*V/RT}$ (s52)

$\delta_{0}=D0*e^{zd*F*V/RT}$ (s53)

where *z* indicates the number of charges associated with the rate and capital letters followed by 0 indicates the value of the rate at 0 mV.

To obtain the time constants of this model we used the Q matrix method [^3^](#_ENREF_3). This method relies on building a singular matrix using the rates of the system; in our case is a 3 x 3 matrix. The eigenvalues of the -Q matrix gives us the inverse of the time constants. For a model like this, also analytical solutions for the time constants have been found [^1^](#_ENREF_1).

Using the rates we can define the equilibrium constants between the states of the collapsed model such that:

$K_{3,2}=\frac{\delta}{\chi}$ (s54)

$K_{2,1}=\frac{\beta}{\alpha}$ (s55)

And the probability of each state would be:

$P_{C1}=\frac{K_{3,2}K_{2,1}}{1+K_{3,2}+K_{3,2}K_{2,1}+K_{3,2}K_{2,1}K_{1,O}P_{C1,0}^{rel}}$ (s56)

$P_{C2}=\frac{K_{3,2}}{1+K_{3,2}+K_{3,2}K_{2,1}+K_{3,2}K_{2,1}K_{1,O}P_{C1,0}^{rel}}$ (s57)

$P_{C3}=\frac{1}{1+K_{3,2}+K_{3,2}K_{2,1}+K_{3,2}K_{2,1}K_{1,O}P_{C1,0}^{rel}}$ (s58)

$P_{O}=\frac{K_{3,2}K_{2,1}K_{1,O}P_{C1,0}^{rel}}{1+K_{3,2}+K_{3,2}K_{2,1}+K_{3,2}K_{2,1}K_{1,O}P_{C1,0}^{rel}}$ (s59)

The term *P_C1,0_^rel^* appears because the open state is in equilibrium with the unbounded *C1* state. Now we can obtain the absolute probability in equilibrium of each substate. This is the probability of each collapsed state multiplied by the relative probability of the substate. As an example, we can write the absolute probability of the first closed unbounded state *P_C1,0_*:

$P_{C1,0}=P_{C1,0}^{rel}P_{C1}$ (s60)

This approach allows us to fit the kinetic and steady-state data simultaneously to our model.

Appendix 2: Detailed description of the global fitting of the allosteric model

Description of the data

The steady-state data includes the G/V curve and inhibition curves (Figure 1 and 2). The kinetic data includes the time constants at different voltages and Ca^2+^ concentrations (Figure 4). The G/V curve was normalized to 0 conductance at -60 mV and for the inhibition curves 0 current was considered as the baseline at -70 mV, the maximal conductance and the minimal inhibition were set as 1. The logarithm of the time constants show homogeneity of variance at all the voltage and Ca^2+^ concentration tested, thus the time constants logarithm were used for the fitting. This property of the variance will be an important supposition for the maximum likelihood-based inference.

Simulation of the data

In order to compare the model output the experimental data, some transformation of the model output is needed.

The *G(V)* curve represents the relative probability of the open state and not the absolute probability. Since at voltages larger than 20 mV the effect of the fast gate starts to be evident we only worked with voltages up to 20 mV. This raises the concern if the maximum tail current was achieved at 20 mV or if in the absence of the fast gate this current will saturate at higher voltages. To overcome this experimental limitation, the *I_max_* was extrapolated from equation 1 and afterward all the *G(V)s* were normalized by the *I_max_* obtained. To simulate the *G(V)* curve the *I_max_* was considered to be the *P_0_* simulated at high voltage of 60 mV and thus we obtain the equation for the simulated *G(V)*:

$G(V)=\frac{P_{O}(V)-P_{O}(-60)}{P_{O}(60)-P_{O}(-60)}$ (s61)

This gives a *G(V)* curve that has a range between 0 and 1 in the interval of voltages between -60 and 60 mV.

The inhibition curves represent the relative probability of the open state at a constant voltage and different Ca^2+^ concentrations, in which the maximum probability is achieved in the absence of Ca^2+^. At a constant voltage, the simulated inhibition curve is given by:

$\frac{I}{I(0Ca^{2+})}=\frac{P_{O}(Ca^{2+})}{P_{O}(0Ca^{2+})}$ (s62)

This gives an inhibition curve that has a maximum of 1 in absence of Ca^2+^.

Since the time constants are directly obtained from the model this output was not further normalized.

Likelihood Estimation

For the likelihood estimation, a likelihood function is needed. This likelihood function varies depending on the underlying probability distribution of the data. Ion channels exist only in two observable conformations, the open and closed conformations, and as such, they can be thought as the output of a Bernoulli process. Since in each recording we have the solution for thousands of Bernoulli processes at different voltages or Ca^2+^ concentration we can think of this as a logistic model. So for the *G(V)* and inhibition curves, we calculated the likelihood function corresponding to the logistic model:

$P(D/\theta)=\prod_{i=1}^{n} \mu_{i}^{x_{i}}*(1-\mu_{i})^{(1-x_{i})}$ (s63)

Where *x_i_* and *μ_i_* correspond to the value of the *ith* observation is the model prediction respectively.

For the fitting of the time constants the values were expressed as logarithms, these values show homogeneity in the variances. Thus the logarithm of the time constants was assumed to have a Gaussian distribution. In this case, the likelihood function corresponding to the Gaussian model was used:

$P(D/\theta)=\prod_{i=1}^{n} \frac{1}{\sqrt{2\pi\sigma^{2}}}*e^{-\frac{\left(x_{i}-\mu_{i}\right)^{2}}{2\sigma^{2}}}$ (s64)

Where σ^2^ corresponds to the variance, this value can be estimated from the sample variance or can be let as a free parameter to be fitted, which was chosen for this work.

Description of the MCMC algorithm

A custom MCMC algorithm based on the Metropolis-Hastings algorithm was utilized [^4^](#_ENREF_4). Uniform priors probabilities were used for the parameters, indicating no prior information or expectations. The voltage dependence of the *G(V)* curve is 2.8, so the prior range for each voltage-dependent transition charge was 0 – 2.8, for the rates at 0 mV the prior range was 0.1 – 100 s^-1^ owing to the slow kinetics of the channel, for the *V1* parameter this was between -200 - 20 mV, for the Kd 0.01 to 10 mM, for c_b_ from 0.5 to 10, for c_f_ from 0.1 to 2 and for the variance of the time constant 0-1.

The basic approach for the MCMC is described as follows:

Being X(0) = {p1(*0*),p2(0),p3(*0*)…..pN(0)} a vector representing the numerical values of all the *N* parameters of the model. We generate randomly a vector *Y* sampling from a *N* dimensional Gaussian distribution with mean *X(0)* and a given covariance matrix Σ. Then the ratio between the posterior probabilities is calculated:

$r=\frac{P(Y/D)}{P(X(0)/D)}=\frac{P(Y)P(D/Y)}{P(X(0))P(D/X(0))}$ (s65)

If *r* is equal or larger than 1 this new set of values is accepted and X(1) = Y, if it's less than 1 then this new set of values is accepted with a probability *r*, otherwise X(1) = X(0). Now, this process is repeated for X(1) and so on.

The first 5*10^4 iterations of the chain were considered as a *burn in* period and consequently discarded. The equilibrium distribution of the chain was utilized to obtain the parameters distribution and thus calculate the most likely values and its errors. From the parameters distributions, 400 sets of parameters were randomly selected to simulate the experimental data and graph the confidence bands.

Model comparison

For determining the probability of a model *M* we can write Bayes’ theorem in the following way:

$P(M/D)=\frac{P(M)P(D/M)}{P(D)}$ (s66)

To compare different models (*M_1_* and *M_2_*) by dividing their posterior probabilities:

$\frac{P(M_{1}/D)}{P(M_{2}/D)}=\frac{P(M_{1})P(D/M_{1})}{P(M_{2})P(D/M_{2})}$ (s67)

This is known as the *odds ratio*. Since the prior probability of the tested models is the same (we do not have a preference of one model over the others) and, the likelihood of a model is equal to the weighted likelihood of the parameter space, we have the expression:$\frac{P(M_{1}/D)}{P(M_{2}/D)}=\frac{P(D/M_{1})}{P(D/M_{2})}=\frac{\int P(D/M_{1},\theta_{1})\partial\theta_{1}}{\int P(D/M_{2},\theta_{2})\partial\theta_{2}}$ (s68)

Where *θ_1_* and *θ_2_* represent a set of parameters in the parameter space of *M_1_* and *M_2_* respectively.

The odds ratio of the models with 2, 3 and 6 binding sites were compared and used to select the most probable model.

Convergence and correlation of MCMC

We tested the convergence of our MCMC using the method developed by Gelman and Rubin[^5^](#_ENREF_5) This method relies on the simulation of several chains. After the burn‑in period, the within chain variance and between chain variance is calculated. The relation between these two is called the potential scale reduction factor (PSRF). Since all the chains are expected to have the same equilibrium distribution, the PSFR is anticipated to be 1 under convergence.

Using different starting values, three chains were simulated and the values for the PSFR along the chain results are shown in supplementary Figure 3. The PSFR has a value near 1 for all the model parameters indicating that the 3 chains converged to similar posterior distributions.

The Pearson's correlation coefficient was calculated for all the model parameters (**Tabe S1**).

References

1. Goldman, L. Gating current kinetics in Myxicola giant axons. Order of the back transition rate constants. *Biophys J* **59**, 574-89 (1991).

2. Cox, D.H., Cui, J. & Aldrich, R.W. Allosteric gating of a large conductance Ca-activated K+ channel. *J Gen Physiol* **110**, 257-81 (1997).

3. Colquhoun, D. & Hawkes, A.G. A Q-Matrix Cookbook. in *Single-Channel Recording* (eds. Sakmann, B. & Neher, E.) 589-633 (Springer US, Boston, MA, 1995).

4. Hastings, W.K. Monte Carlo sampling methods using Markov chains and their applications. *Biometrika* **57**, 97-109 (1970).

5. Gelman, A. & Rubin, D.B. Inference from Iterative Simulation Using Multiple Sequences. *Statistical Science* **7**, 457-472 (1992).

**Supplementary Figures and Table**


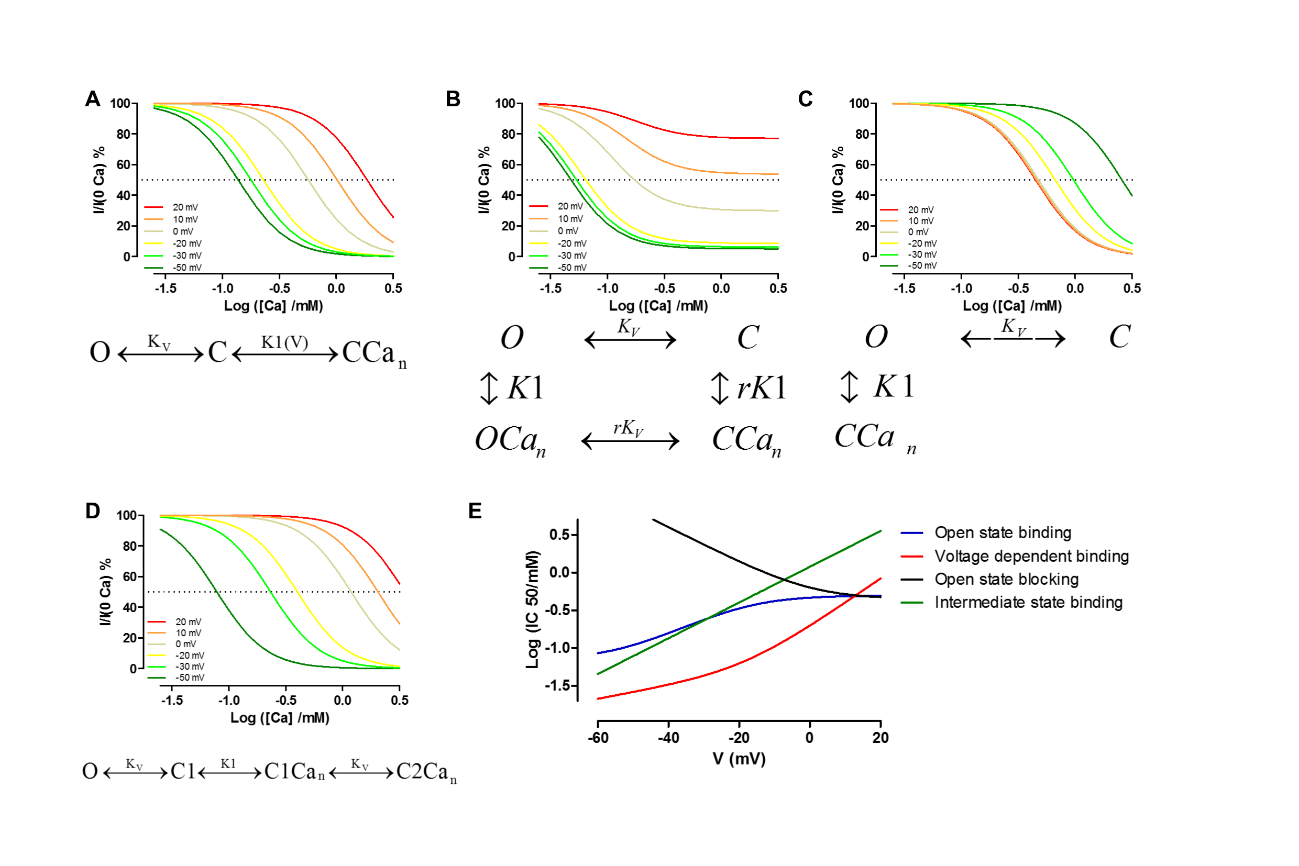


**Supplementary figure 1: Inhibition curves of alternative models.** (**A**) Ca^2+^ inhibition curve at different voltages using the linear Hill model with a voltage-dependent binding of Ca^2+^ (zδ = 0.2). (**B**) Ca^2+^ inhibition curve at different voltages using the squared model in which Ca^2+^ binds to the open state with 20 times lower affinity than to the closed state. (**C**) Open state binding of Ca^2+^ to generate an occluded or blocked state. (**D**) Binding of Ca^2+^ to an intermediate closed state. (**E**) Simulated Log *IC50* curves for the different models. In figures **A-D** the kinetic scheme of the model is presented at the bottom of the inhibition curves.

**
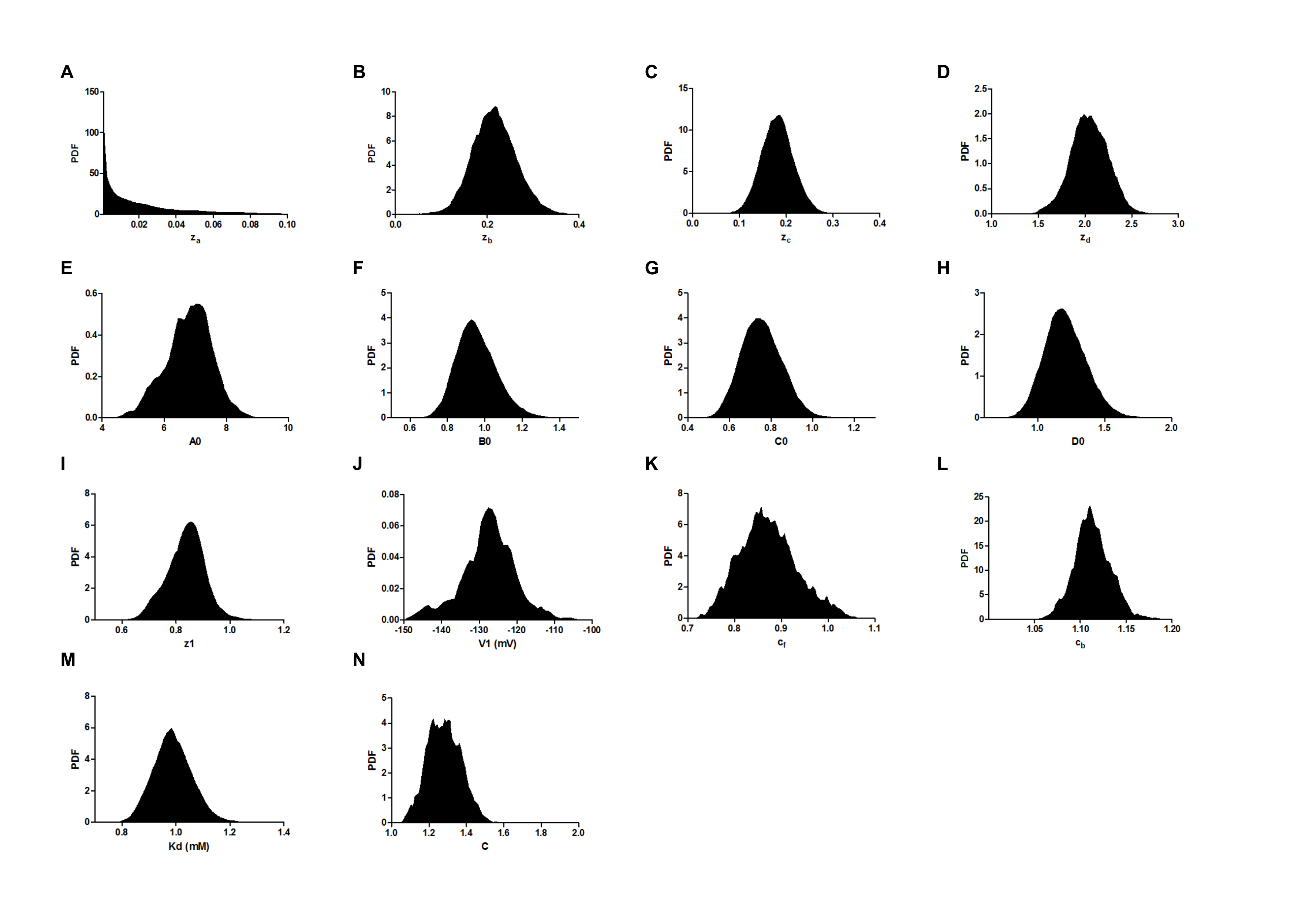
**

**Supplementary figure 2: Parameter distributions for the allosteric model.** Histograms of the posterior probability of the different parameters of the allosteric model.

**Supplementary figure 3: Convergence of parameters in parallel chains.** Calculated potential scale reduction factor (PSRF) for each parameter along iterations. The PSRF was calculated from three parallel MCMC chains with different starting points, after the burn-in period.

**Supplementary table :** **Correlation Matrix of the posterior distribution of the model.**

|  | **za** | **zb** | **zc** | **zd** | **A0** | **B0** | **C0** | **D0** | **z1** | **V1** | **Kd** | **cb** | **cf** |
| --- | --- | --- | --- | --- | --- | --- | --- | --- | --- | --- | --- | --- | --- |
| **za** | 1 | 0.46 | 0.33 | -0.3 | -0.48 | -0.29 | -0.42 | 0.23 | 0.48 | 0.04 | -0.31 | 0.4 | 0.03 |
| **zb** |  | 1 | 0.4 | -0.43 | -0.8 | -0.21 | -0.65 | 0.27 | 0.8 | -0.21 | -0.54 | 0.75 | 0.19 |
| **zc** |  |  | 1 | -0.15 | -0.49 | -0.51 | -0.91 | 0.2 | 0.47 | 0.02 | -0.32 | 0.45 | 0.11 |
| **zd** |  |  |  | 1 | 0.41 | 0.17 | 0.27 | -0.18 | -0.43 | 0.05 | 0.38 | -0.34 | -0.06 |
| **A0** |  |  |  |  | 1 | 0.49 | 0.72 | -0.38 | -0.79 | 0.29 | 0.63 | -0.83 | -0.32 |
| **B0** |  |  |  |  |  | 1 | 0.57 | -0.6 | -0.52 | 0.06 | 0.35 | -0.49 | -0.19 |
| **C0** |  |  |  |  |  |  | 1 | -0.31 | -0.7 | 0.27 | 0.43 | -0.76 | -0.13 |
| **D0** |  |  |  |  |  |  |  | 1 | 0.53 | -0.12 | -0.32 | 0.37 | -0.03 |
| **z1** |  |  |  |  |  |  |  |  | 1 | -0.83 | -0.51 | 0.79 | 0.04 |
| **V1** |  |  |  |  |  |  |  |  |  | 1 | -0.11 | -0.74 | 0.03 |
| **Kd** |  |  |  |  |  |  |  |  |  |  | 1 | -0.42 | -0.46 |
| **cb** |  |  |  |  |  |  |  |  |  |  |  | 1 | 0.25 |
| **cf** |  |  |  |  |  |  |  |  |  |  |  |  | 1 |
